# Strong Light Confinement in Metal-Coated Si Nanopillars: Interplay of Plasmonic Effects and Geometric Resonance

**DOI:** 10.1186/s11671-017-1932-0

**Published:** 2017-02-27

**Authors:** Sujung Kim, Eunah Kim, Yeon Ui Lee, Eunkyo Ko, Hyeong-Ho Park, Jeong Weon Wu, Dong-Wook Kim

**Affiliations:** 1 Department of Physics, Ewha Womans University, Seoul, 120-750 Korea; 2 Department of Chemistry and Nano Science, Ewha Womans University, Seoul, 120-750 Korea; 3 Device Platforms Lab., Device Engineering Labs., Korea Advanced Nano Fab Center (KANC), Suwon, 443-270 Korea

**Keywords:** Si, Nanopillar array, Localized surface plasmon, Surface plasmon polariton, Mie resonance, 88.40.H-, 88.40.jp, 81.07.Gf

## Abstract

We investigated the influence of metal coating on the optical characteristics of Si nanopillar (NP) arrays with and without thin metal layers coated on the sample surface. The reflection dips of the metal-coated arrays were much broader and more pronounced than those of the bare arrays. The coated metal layers consisted of two parts—the metal disks on the Si NP top and the holey metal backreflectors on the Si substrate. The Mie-like geometrical resonance in the NPs, the localized surface plasmons in the metal disks, and the propagation of surface plasmon polariton along the backreflector/substrate interface could contribute to the reflection spectra. Finite-difference time-domain simulation results showed that the interplay of the plasmonic effects and the geometric resonance gave rise to significantly enhanced light confinement and consequent local absorption in the metal-Si hybrid nanostructures.

## Background

Semiconductor nanostructure arrays exhibit unique optical responses that cannot appear in their bulk counterparts. Multiple scattering, graded refractive index, diffraction, Fabry–Perot interference, and Mie-like geometrical resonance enable nanostructured semiconductors to dramatically suppress optical reflection and strongly concentrate light in a local region [1–9]. Enhanced light scattering and absorption at resonance wavelengths can be used for all-dielectric color filtering [1–3], enhanced Raman scattering [4, 5], and color imaging [6]. Broadband antireflection effects and environment-sensitive optical responses are beneficial for high-efficiency solar cells [7, 8] and sensors [9], respectively. Semiconductor nanostructures have advantages of competitive cost, well-established fabrication processes, and improved long-term reliability.

Operation of semiconductor devices usually requires metal electrodes, which are used for application of bias voltage, injection of charge carriers, and detection of current flow, and nanostructured devices are not exempt. When metal electrodes have nanoscale dimensions, surface plasmons (SPs)—collective oscillation of the conduction electrons—can enhance scattering and absorption cross section of the metal nanostructures along with spatially confined light at the resonance [10]. Synergistic effects of the plasmonic and geometrical optical resonances are expected to occur in metal-semiconductor hybrid nanostructures, which could exhibit intriguing optical characteristics [11–15].

In this work, we fabricated Si nanopillar (NP) arrays and investigated their optical properties. Deposition of thin metal (Ag and Au) layers in high vacuum allowed us to form metal disks at the top of the Si NP and backreflectors on the Si substrate. The geometric optical resonance, the localized SP in the disk, and the propagation of surface plasmon polaritons (SPPs) along the backreflector/substrate interface can contribute to the optical response of the metal-coated Si NP arrays. Not only each of these issues, but also the interplay among them, can influence the optical characteristics of metal-coated Si NP arrays. Reflection spectra in the visible-to-near-infrared regime were obtained, and the physical origins of the spectra were discussed based on optical simulations.

## Methods

**Fig. 1:**
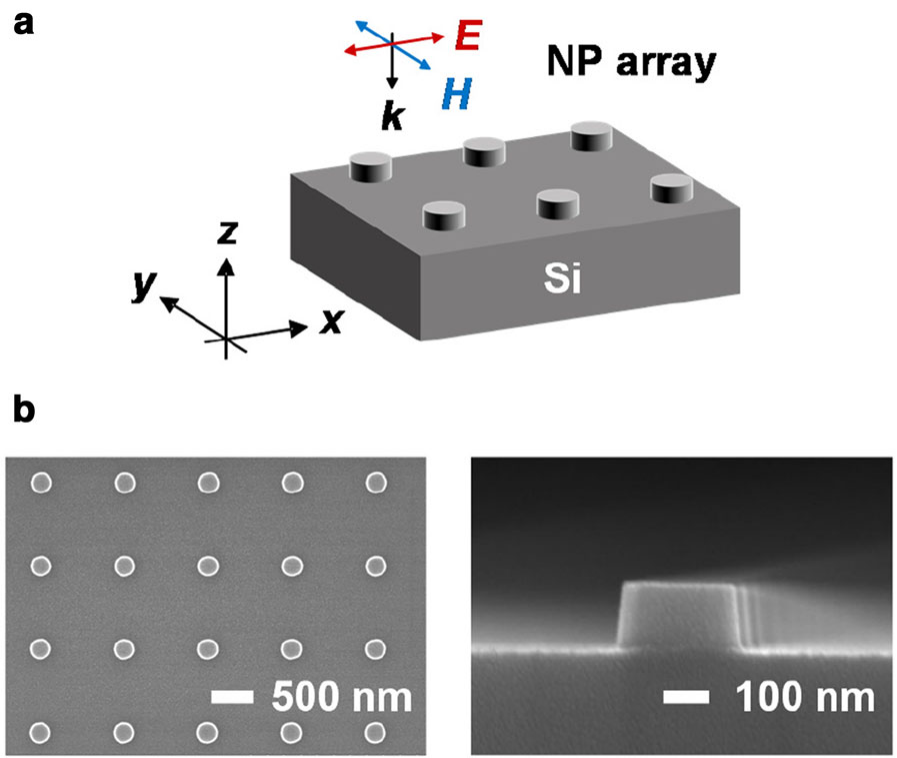
**a** Schematic diagram and **b** top-down and cross-sectional scanning electron microscope images of the bare Si NP array

Square arrays of Si NPs (diameter 250 nm, height 150 nm, period 1 μm, and area 1 × 1 mm^2^) were fabricated by electron beam lithography (EBL) and dry etching, as shown in Fig. 1a, b. An n-type Si wafer with doping concentration of 10^17^ cm^−3^ was coated with chemically amplified negative tone resist (NEB; Sumitomo Chemical), and then exposed to 100-keV electron beams using an EBL system (JBX9300FS, JEOL). After developing, the wafer with the resist pattern was etched using a deep reactive ion etcher (AMS200, Alcatel) and regular NP arrays were obtained, as shown in Fig. 1b. On the Si NP array, Ag and Au thin films were deposited using an electron beam evaporator. Optical reflection spectra of the bare and metal-coated NP arrays, in visible and near-infrared wavelength range, were obtained using a homemade microspectrometer with a halogen lamp as a light source [12]. The incident beam with a spot size of 400 μm is normally incident to the NP array, and the reflected light was collected to a spectrometer via an optical fiber.

To investigate the optical properties of the bare and metal-coated Si NP arrays, finite-difference time-domain (FDTD) simulations were performed using a commercial software package (Lumerical Solutions, ver. 8.12.501). For the reflection spectra calculations, the size of the periodic simulation domain was set to 1.0 × 1.0 × 14 μm^3^, and a broad wavelength (*λ*) range (400–1100 nm) of a plane wave light source was normally incident to the samples (Fig. 1a). Periodic boundary conditions were used along the in-plane directions, and perfectly matched layers were used as the radiation boundary condition at the top and bottom of the simulated structure. Monitors for collecting reflected light were placed above the light source. The optical constants of crystalline Si, Ag, and Au were taken from Palik [16].

## Results and Discussion

**Fig. 2:**
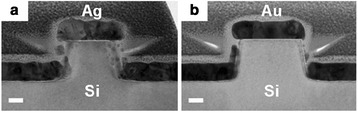
Cross-sectional TEM images of the NP arrays coated with **a** Ag and **b** Au thin films (*scale bar* 50 nm)

Figure 2a, b shows transmission electron microscope (TEM) images of the Ag- and Au-coated Si NP arrays, respectively. The evaporation of the metal thin films was performed in a high-vacuum conditions (pressure <10^−6^ Torr), where the mean free path was much longer than the source-to-sample distance of our evaporator. As a result, the metal thin films were barely deposited on the side walls of the NPs. Thus, the metal thin layers formed two parts—disks on the NP and holey backreflectors on the substrate, as shown in Fig. 2a, b. The TEM images also showed that the Si surface is covered by very thin native oxide layers (thickness ~20 Å).

**Fig. 3:**
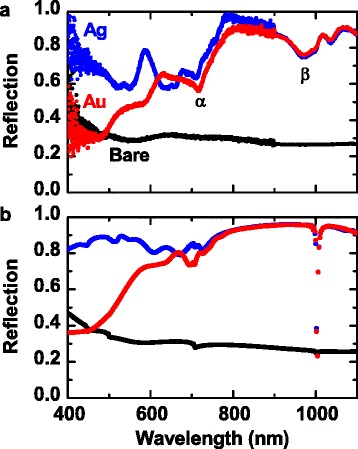
**a** Experimental and **b** calculated reflection spectra of the bare and metal-coated Si NP arrays

Figure 3a, b shows the experimental and calculated reflection spectra of the bare and metal-coated Si NP arrays. The bare sample has no clear features and relatively low reflectance in the whole wavelength range. The overall reflected light intensity from the metal-coated samples is much larger than that from the bare sample, as both the Ag and Au thin films work as good mirrors in the measured wavelength range. The reflection from the Au-coated sample drops at *λ* < 600 nm due to the interband transition of Au [10]. The experimental reflection spectra of the metal-coated NPs have broad dips at *λ* ~700 and ~1000 nm, indicated by α and β in Fig. 3a. As shown in Fig. 3b, the FDTD-calculated spectra look similar to the experimental results for the three kinds of samples, when the overall magnitude and local dip positions are compared. It can be also noted that the calculated reflection for the bare sample has very weak dips near α and β. All the dips in the measured data are much broader and more pronounced than those in the simulation data. In the calculation studies, the geometric parameters of the NPs were taken from typical SEM and TEM images, and uniform native oxide layers were considered. Thus, the discrepancies between the experimental and calculation results could be attributed to the somewhat irregular geometric configuration and rough surface of the fabricated NP arrays. In particular, the calculated β, observed at *λ* ~ 1000 nm (the period of the NP array), is very deep and narrow, indicating that this dip could originate from the Wood anomaly [17].

**Fig. 4:**
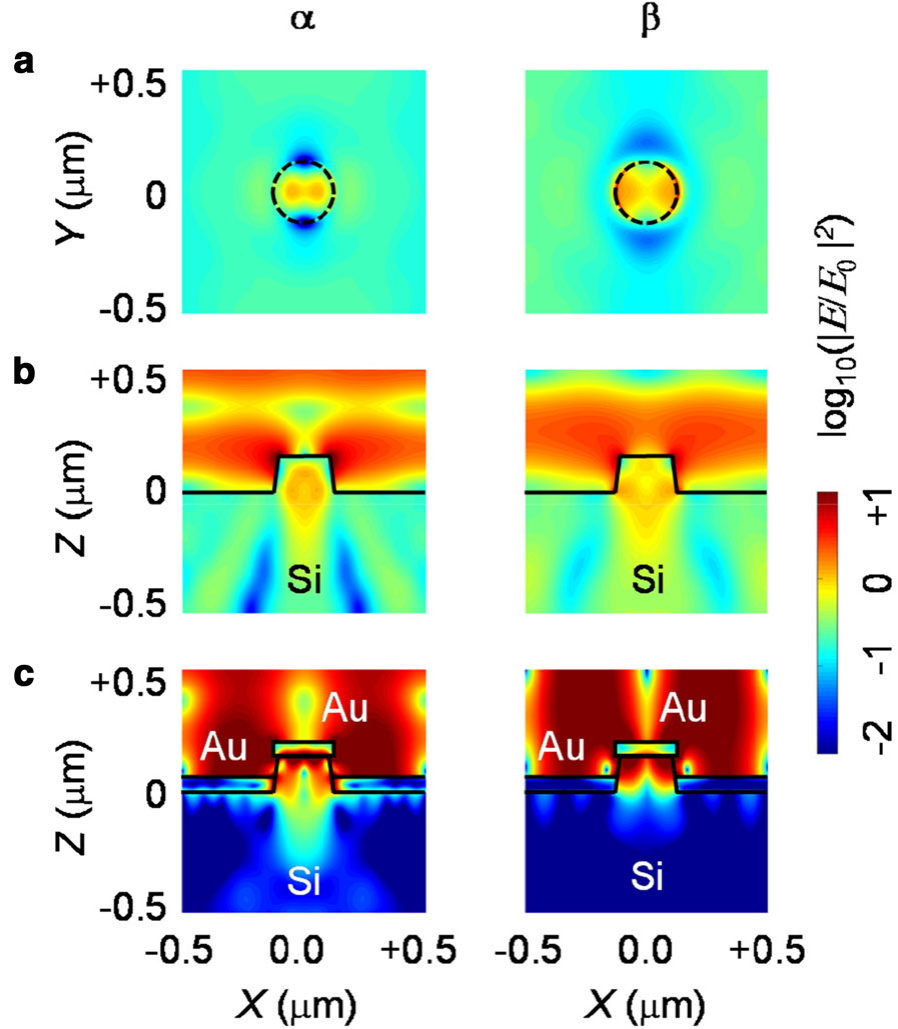
**a**, **b** The simulated electric field intensity distributions for the bare Si NP and **c** the Au-coated NP. Left and right distributions correspond to the results obtained at α and β, respectively

Figure 4a–c shows the simulated electric field intensity distributions in the bare and metal-coated NPs under illumination of monochromatic light. The intensity profiles of the bare NP array in Fig. 4a, b show that the incident light is strongly concentrated in and around the NPs at α and β. Groep and Polman systematically investigated the geometric optical resonance of semiconductor nanoresonators on substrates [7]. According to their work, α and β can be assigned to the merged electric/magnetic dipole (ED/MD) mode and the magnetic quadrupole (MQ) mode, respectively. Figure 4b also shows that the NPs scatter large amount of the incoming light to the underlying substrates, as the presence of the large refractive index Si can modify the resonant modes [7]. Such interaction is often used to enhance the optical absorption of Si for photovoltaic applications [8].

The local confinement of the incoming light in the NPs becomes much more significant in the metal-coated NPs compared with the bare NPs, as shown in Fig. 4b, c. It has been reported that metal nanodisks on top of Si nanostructures can trap light efficiently in nanostructures, resulting in pronounced and narrow reflection dips [14, 15]. With the metal disk on top of the Si nanostructures, the refractive index contrast at the air/metal/Si interface increased as compared with that at the air/Si interface. The consequent stronger Fabry–Perot resonance can enhance the spectral dips [14]. Very large field intensity is observed at the Au disk/Si NP interface as well as in the NP (Fig. 4c). Such field confinement was not seen for the Au disks on a flat Si substrate (data not shown here). This suggests that the local field enhancement cannot be solely attributed to the localized SP effects. Dhindsa and Saini also noted similar field localization at their Al-disk/Si-nanowire interface [14]. They claimed that the geometric resonance and antireflection properties of the nanostructures could cause the electric field confinement at the metal disk/Si-nanostructure interface.

Figure 4c also shows that the field intensity in the substrate under the holey backreflector is very weak, as the metal layers block the incident light. In contrast, a clear field pattern can be seen at the Au backreflector/Si interface. The field looks like propagating along the interface, which may indicate SPP excitation [10–12]. The backreflector has a periodic hole array, which provides in-plane direction momentum to SPPs [10]. Thus, SPPs with specific energies, as determined by the dispersion relation, can couple with photons and vice versa. Such coupled SPP excitation could contribute to the spectral response of our metal-coated Si NP array.

Kumar et al. reported interesting optical properties of polymer (negative tone electron beam resist HSQ) NP arrays with top metal disks and holey backreflectors, similar to our samples [18]. They achieved full-color generation by varying the size and separation of their NPs. Strong wavelength-selective scattering features could be attributed to plasmonic and Fano resonances. Their NPs consisted of a polymer with a relatively low refractive index. In contrast, our Si NPs can exhibit Mie-like resonance in visible and near-infrared wavelength ranges because of the large refractive index of Si (see Fig. 4a, b). Therefore, both the plasmonic effects in the metal layers and the geometric resonance in the Si NPs should be considered to explain the optical properties of our metal-coated Si NP arrays.

**Fig. 5:**
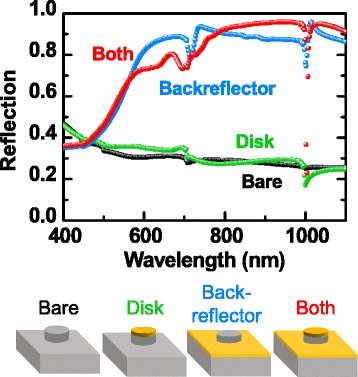
The simulated reflection spectra of four kinds of Si NP arrays, of which configurations are schematically illustrated

Figure 5 shows the simulated reflection spectra of four kinds of Si NP arrays and schematic illustrations of their configurations. The reflection of the NP array with only Au disks at the top (this sample will be indicated by “disk” hereafter) is not much different from that of the bare NP array. This suggests that the localized SP effects of the Au top disks alone cannot notably alter the spectral response of the sample. The NP array with only a Au holey backreflector (hereafter indicated by “backreflector”) has much larger reflection compared with the bare sample and disk, as a large portion of the surface area is covered by the highly reflective Au thin film. Furthermore, the reflection dips of the backreflector are much more pronounced, compared with those of the bare sample and disk. As discussed above, SPPs can be coupled to incident photons at the Au/Si interface in the backreflector. The coupled SPP can be excited at several wavelengths from 700 to 1000 nm, considering the dispersion relation (not shown here) and the period of the hole array. Figure 4c shows the electric field pattern confined at the interface of the Si substrate and the Au backreflector, as aforementioned. The Si NPs may scatter the propagating SPPs and consequently emit light. This can further raise the field intensity in the NPs, in addition to the geometric resonance-induced field confinement [11]. Considering long penetration depths of Si (i.e., 40 μm at *λ* = 900 nm), the light concentration in the tiny NP is enormous (Fig. 4c). As a result, the reflection spectra of the backreflector have notable local minima, at which wavelengths of the SP effects and the Mie-like resonance notably increase the optical absorption in the NPs. The reflection spectra of the NP array with the metal disk at the NP top and the holey backreflector (hereafter, indicated by “both”) are more or less similar to those of the backreflector. At *λ* ~ 1000 nm, somewhat asymmetric dips can be found, which may originate from the Fano resonance, i.e., interference between the geometric resonance mode (enhanced by SPPs) and Wood anomaly [19].

## Conclusions

We fabricated square arrays of Si NPs and investigated the influence of thin metal (Ag and Au) layers on their optical reflection spectra. The reflection dips of the metal-coated NP arrays were broad and pronounced, whereas the dips of the bare array were very weak. FDTD simulations showed that the dip positions corresponded to the geometric optical resonance wavelengths of the Si NPs. The metal-coated NP arrays consisted of the Si NPs, the metal disks on the NP top, and the holey metal backreflectors on the substrate surface. The simulation results revealed that the metal disk alone cannot enhance the reflection dip much, and the SPP excitation along the backreflector/substrate interface was crucial in the optical response of the coated array. The comparative experimental and calculation works clearly suggest that the interplay of the SP effects and the geometric resonance significantly enhanced light confinement in the Si NPs and contributed to pronounced reflection dips in our metal-coated Si NP arrays.

## Abbreviations

EBL: Electron beam lithography
ED: Electric dipole
FDTD: Finite-difference time-domain
MD: Magnetic dipole
MQ: Magnetic quadrupole
NEB: Negative tone resist
NP: Nanopillar
SP: Surface plasmon
SPP: Surface plasmon polariton
TEM: Transmission electron microscope
